# Stimulus Complexity and Mouse Strain Drive Escalation of Operant Sensation Seeking Within and Across Sessions in C57BL/6J and DBA/2J Mice

**DOI:** 10.3389/fnbeh.2019.00286

**Published:** 2020-01-10

**Authors:** Price E. Dickson, Guy Mittleman

**Affiliations:** ^1^The Jackson Laboratory, Bar Harbor, ME, United States; ^2^Department of Psychological Science, Ball State University, Muncie, IN, United States

**Keywords:** addiction, substance use, reward, novelty seeking, sensitization, habituation, systems genetics, BXD

## Abstract

Sensation seeking is a heritable trait that is genetically correlated with substance use; the shared genetic mechanisms underlying these traits are largely unknown. The relationship of sensation seeking and substance use has practical importance because discovering genes that drive sensation seeking can reveal genes driving substance use, and quantification of sensation seeking in mice is higher throughput and less technically challenging than quantification of volitional drug use. In order to fully understand the genetic mechanisms driving sensation seeking, it is critical to first understand the nongenetic factors driving sensation seeking. In the present study, we used the operant sensation seeking paradigm to assess the effects of stimulus complexity on sensation seeking in C57BL/6J and DBA/2J mice. These strains are the founders of the BXD recombinant inbred mouse panel which enables the discovery of genes driving phenotypic variation. This study led to four principal conclusions. First, all sensory stimuli used in the study, regardless of complexity or number of stimulus modalities, served as reinforcers for C57BL/6J and DBA/2J mice. Second, for both C57BL/6J and DBA/2J mice, sensation seeking for a high complexity sensory stimulus was significantly greater than sensation seeking for a low complexity sensory stimulus. Third, for both C57BL/6J and DBA/2J mice, sensation seeking escalated significantly within-session when a multimodal sensory stimulus of medium or high complexity was used but not when a unimodal sensory stimulus of low complexity was used. Finally, both the magnitude of sensation seeking and the magnitude of within-session escalation of sensation seeking were significantly greater in mice from the DBA/2J strain relative to mice from the C57BL/6J strain. Collectively, these findings indicate that stimulus complexity and genetic background drive escalation of operant sensation seeking within and across sessions, and that the BXD recombinant inbred mouse panel can be used to discover the genetic mechanisms underlying these phenomena.

## Introduction

Animal behavior is reinforced by sensory stimuli, and the biological mechanisms driving sensation seeking are shared with those driving substance use (Zuckerman, 1986; Piazza et al., 1990; Olsen and Winder, 2009; Belin and Deroche-Gamonet, 2012; Flagel et al., 2014; Dickson et al., 2015, 2016, 2019). This phenomenon is theoretically important because it illustrates that abused substances hijack the most fundamental mechanisms motivating animal behavior. The relationship of sensation seeking and substance use also has practical importance: the discovery of genes driving sensation seeking can lead us to genes driving substance use (Dickson et al., 2019). The genetic mechanisms driving substance use and sensation seeking are largely unknown.

In order to fully understand the genetic mechanisms that underlie sensation seeking, it is critical to first understand the nongenetic factors that underlie sensation seeking. In this regard, little is known about the relationship between sensory stimulus characteristics and sensation seeking behavior. Some important questions are: (1) what stimulus characteristics enable sensory stimuli to serve as reinforcers; (2) how does experimentally manipulating these stimulus characteristics influence sensitization and habituation to sensory reinforcement; and (3) how are these phenomena influenced by genetic factors? Answering these questions will move us closer to discovering the specific genes and networks which drive sensation seeking and understanding how they are hijacked by abused substances.

In the present study, we used the operant sensation seeking (OSS) paradigm (Dickson et al., 2019) to quantify sensation seeking in C57BL/6J and DBA/2J mice. The C57BL/6J and DBA/2J strains are the founders of the BXD recombinant inbred panel (Peirce et al., 2004; Ashbrook et al., 2019) which enables the discovery of genetic mechanisms driving phenotypic variation (Dickson et al., 2016, 2019; Parker et al., 2017). We quantified OSS in C57BL/6J and DBA/2J mice using sensory stimuli of a low, medium, or high complexity. A control group for which sensory stimuli were not delivered following a lever press was also tested. Mice were tested under these experimental conditions across four 15-min bins for 26 sessions. Using these data, we assessed the influence of stimulus complexity and mouse strain on OSS both across sessions and within sessions.

## Materials and Methods

### Subjects

Male and female C57BL/6J mice (stock number: 000664) and DBA/2J mice (stock number: 000671) were purchased from The Jackson Laboratory (Bar Harbor, ME, USA). A single male and a single female of the same strain were housed together in standard cages in ventilated racks in the Animal Care Facility in the Department of Psychology at the University of Memphis. Male offspring from these breeder pairs were used as experimental subjects. Experimental subjects were weaned at 4 weeks of age and housed in same-sex groups of 3–5 in standard mouse pens. Mice were individually housed at the start of the experiment which began when mice were ~12 weeks of age. Mice were maintained in a temperature-controlled environment (21 ± 1°C) on a 12:12 light:dark cycle (lights on at 08:00). Mice had free access to food and water throughout the experiment.

### Apparatus

OSS data were collected using 12 Med Associates operant conditioning chambers (307W) each enclosed in a sound-attenuating cubicle (ENV-022MD). Two retractable response levers (ENV-310W) were mounted on the front wall of each chamber. A stimulus light (ENV-321W) was mounted above each lever. A house light (ENV-315W) with bulb (Chicago Miniature Lighting, LLC., Novi, MI, USA; CM1829) was centrally mounted on the rear wall of each chamber. Operant conditioning chambers were controlled by two Lafayette Instruments (Lafayette, IN, USA) BNC MK I control units. The program used to run the OSS protocol was written in-house using the Campden BNC Control software (version 1.23).

### Behavioral Testing and Experimental Groups

#### OSS

Mice were tested for 26 sessions. Each session lasted for 60 min. Testing occurred once per day, at the same time, 7 days per week. Each session began with the illumination of the house light and extension of the two response levers. For all mice, the right lever was defined as the active lever and the left lever was defined as the inactive lever. Mice were tested on a fixed ratio 1 (FR1) operant conditioning schedule in which the consequences of a single active lever press varied as a function of experimental condition (Table 1): For mice in the *No stimuli* condition, an active lever press had no consequences. For mice in the *Lights* condition, the *Levers* condition, and the *Lights + Levers* condition, an active lever press delivered a stimulus of low, medium, or high complexity, respectively (stimuli are described in detail in the next section). Throughout the entire session, inactive lever presses were recorded but had no consequences.

**Table 1:** Experimental conditions and associated sensory stimuli used in the operant sensation seeking paradigm.

Stimulus condition	Stimulus complexity	Sensory modalities	Components of the sensory stimulus			Sample size (*N* = 91)	
			Visual	Auditory	Tactile	C57BL/6J (*n* = 45)	DBA/2J (*n* = 46)
No stimuli	-	0	-	-	-	12	12
Lights	Low	1	See lights flash	-	-	12	11
Levers	Medium	3	See levers retract	Hear levers retract	Feel levers retract	10	11
Lights + Levers	High	3	See lights flash and levers retract	Hear levers retract	Feel levers retract	11	12

#### Sensory Stimuli

When mice in the *Lights* condition pressed the active lever, the house light was switched off and the stimulus lights above the active and inactive levers were flashed (i.e., rapidly switched on and off). Flash duration (1, 2, 4, or 8 s) and the number of flashes per second (5, 2.5, 1.25, or 0.625) were randomized independently across stimulus presentations. The house light was switched back on after stimulus light flashing was complete; the next stimulus presentation (i.e., flashing of stimulus lights) could not be delivered until the house light had been switched back on. Both active and inactive lever presses were recorded at all times during the session.

When mice in the *Levers* condition pressed the active lever, both the active and inactive levers were retracted; levers were extended following 750 ms. The sound intensity of lever retraction was 68 dBs. The next stimulus presentation (i.e., lever retraction) could not be delivered until 1, 2, 4, or 8 s had elapsed following the previous lever retraction (randomized across stimulus presentations). This requirement ensured that the maximum number of stimulus presentations that could be self-administered during a session was equal for the *Lights* condition and the *Levers* condition. Both active and inactive lever presses were recorded at all times during the session.

When mice in the *Lights + Levers* condition pressed the active lever, the house lights flashed as described for mice in the *Lights* condition, and the levers were retracted as described for mice in the *Levers* condition. At the beginning of each stimulus presentation, levers were retracted and lights began flashing simultaneously. The house light was switched back on after stimulus light flashing was complete, and the next stimulus presentation (i.e., flashing of stimulus lights with lever retraction) could not be delivered until this had occurred. This requirement ensured that the maximum number of stimulus presentations that could be self-administered during a session was equal for all three conditions in the study. In all conditions, both active and inactive lever presses were recorded at all times.

#### Stimulus Complexity

As described in Table 1, we characterized the flashing of stimulus lights in the absence of other sensory stimuli as a low complexity stimulus because light stimulates a single sensory modality (i.e., mice could see lights flash, but not hear or feel lights flash). We characterized lever retraction as a medium complexity stimulus because lever retraction stimulates multiple sensory modalities (i.e., mice could see, hear, and feel levers retract). We characterized the combination of lever retraction and flashing stimulus lights as a high complexity stimulus because this combination stimulates multiple sensory modalities and the total number of stimulus events is greatest relative to the other conditions (i.e., mice could see lights flash, see levers retract, hear levers retract, and feel levers retract).

### Statistical Methods

Number of active lever presses and the number of inactive lever presses were collected on each of the four 15-min time bins on each of the 26 sessions; these data were used in statistical analyses which were conducted using SPSS version 24. Analysis of variance (ANOVA) was used to assess performance on the OSS assay. The SPSS GLM command was used to conduct three-way repeated-measures ANOVAs and two-way ANOVAs. The assumption of homogeneity of variance across groups and sessions was assessed using Mauchly’s test of sphericity. The Huynh–Feldt correction was used when this assumption was violated. When conducting ANOVAs, *post hoc* tests associated with the highest order interaction that reached statistical significance were reported. If an interaction did not reach statistical significance, *post hoc* tests associated with statistically significant main effects were reported. To adjust for multiple comparisons during *post hoc* testing, Fisher’s least significant difference (LSD) procedure was used. *Post hoc* tests were conducted during the process of performing ANOVAs by using the EMMEANS subcommand with the COMPARE (factor) ADJ (LSD) specification.

## Results

### OSS Is Enhanced by Stimulus Complexity in C57BL/6J and DBA/2J Mice

**Figure 1:**
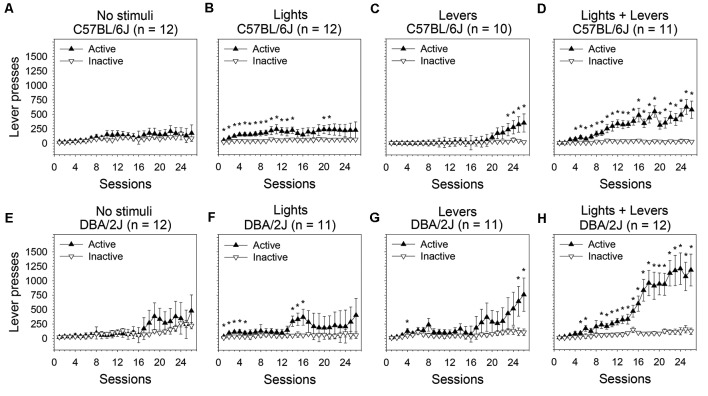
Operant sensation seeking (OSS) is enhanced by stimulus complexity in C57BL/6J and DBA/2J mice. Following an active lever press in an operant conditioning chamber, male C57BL/6J and DBA/2J mice received one of the following stimulus presentations: no sensory stimuli, flashing lights, retracting levers, or the combination of flashing lights and retracting levers. The relationship between active lever pressing and inactive lever pressing varied significantly across sessions as a function of stimulus complexity as indicated by a statistically significant three-way interaction of these factors (three-way analysis of variance, ANOVA) in both C57BL/6J mice (*F*_(75,1025)_ = 1.89, *p* = 0.04) and DBA/2J mice (*F*_(75,1050)_ = 2.08, *p* = 0.04). *Post hoc* tests (Fisher’s least significant difference, LSD) were performed to characterize these relationships: **(A,E)** In the *No stimuli* condition, neither C57BL/6J nor DBA/2J mice distinguished the active lever from the inactive lever. **(B,C,D,F,G,H)** In all conditions in which sensory stimuli were used as a reinforcer, both C57BL/6J and DBA/2J mice pressed the active lever significantly more than the inactive lever on at least some sessions and exhibited an acquisition curve; the most robust response for both strains was observed in the *Lights + Levers* condition. *Sessions on which the number of active lever presses was significantly greater (*p* < 0.05) than the number of inactive lever presses within strain and stimulus condition. Data points represent means. Error bars represent standard errors.

To assess performance on the OSS assay across sessions, we performed a three-way ANOVA. The number of lever presses was used as the dependent variable. The three independent variables were stimulus complexity (levels: *No stimuli, Lights, Levers, Lights + Levers*), session (levels: 1–26), and lever (levels: *active, inactive*). Stimulus complexity was a between-subjects variable whereas session and lever were within-subjects variables. This analysis was performed separately for C57BL/6J mice and DBA/2J mice, and the results for each strain are reported below and illustrated in Figure 1.

#### C57BL/6J Mice

In C57BL/6J mice (Figures 1A–D), we observed statistically significant main effects of stimulus complexity (*F*_(3,41)_ = 2.92, *p* = 0.04), session (*F*_(25,1025)_ = 9.58, *p* = 0.000001), and lever (*F*_(1,41)_ = 32.13, *p* = 0.000001). Most importantly, the three-way interaction of these factors was statistically significant (*F*_(75,1025)_ = 1.89, *p* = 0.04) indicating that the relationship between the active lever and the inactive lever changed across sessions as a function of stimulus complexity. Because the three-way interaction was statistically significant, we performed *post hoc* tests (Fisher’s LSD) to assess the differences between the active and inactive lever on all 26 sessions at each of the four levels of stimulus complexity.

C57BL/6J mice pressed the active lever significantly more than the inactive lever on 16 sessions in the *Lights* condition (Figure 1B), four sessions in the *Levers* condition (Figure 1C), and 23 sessions in the *Lights + Levers* condition (Figure 1D; *p* < 0.05 for all tests). As expected, the number of lever presses on the active lever and the inactive lever in the *No stimuli* condition did not differ significantly for C57BL/6J mice (Figure 1A). These data indicate that: (1) active lever pressing of C57BL/6J mice was reinforced independently by the flashing of stimulus lights and the retraction of response levers; and (2) the most robust response was observed when these stimuli were combined.

#### DBA/2J Mice

In DBA/2J mice we observed statistically significant main effects of session (*F*_(25,1050)_ = 12.52, *p* = 0.000003) and lever (*F*_(1,42)_ = 18.27, *p* = 0.0001). The main effect of stimulus complexity approached significance (*F*_(3,42)_ = 2.49, *p* = 0.07). Most importantly, the three-way interaction of these factors was statistically significant (*F*_(75,1050)_ = 2.08, *p* = 0.04) indicating that the relationship between active lever pressing and inactive lever pressing changed across sessions as a function of stimulus complexity. Because the three-way interaction was statistically significant, we performed *post hoc* tests (Fisher’s LSD) to assess the difference between the active and inactive lever on all 26 sessions for each of the four levels of stimulus complexity.

DBA/2J mice pressed the active lever significantly more than the inactive lever on eight sessions in the *Lights* condition (Figure 1F), three sessions in the *Levers* condition (Figure 1G), and 20 sessions in the *Lights + Levers* condition (Figure 1H; *p* < 0.05 for all tests). As expected, the number of lever presses on the active lever and the inactive lever in the *No stimuli* condition did not differ significantly for DBA/2J mice (Figure 1E). Collectively, these data indicate that: (1) active lever pressing of C57BL/6J mice and DBA/2J mice was reinforced independently by the flashing of stimulus lights and the retraction of response levers; and (2) the most robust response for both strains was observed when these stimuli were combined.

### The Magnitude of OSS Varies With Mouse Strain and Is Enhanced by Stimulus Complexity

To assess the effects of stimulus complexity and mouse strain on the magnitude of OSS following 26 days of testing, we performed a two-way ANOVA. The number of active lever presses (mean of final four sessions) was used as the dependent variable. The two independent variables were stimulus complexity (levels: *No stimuli, Lights, Levers, Lights + Levers*) and mouse strain (levels: C57BL/6J, DBA/2J). Stimulus complexity and strain were between-subjects variables. We observed statistically significant main effects of stimulus complexity (*F*_(3,83)_ = 3.89, *p* = 0.01) and mouse strain (*F*_(1,83)_ = 4.34, *p* = 0.04). The two-way interaction of stimulus complexity and mouse strain was not statistically significant (*F*_(3,83)_ = 0.75, *p* = 0.52).

**Figure 2:**
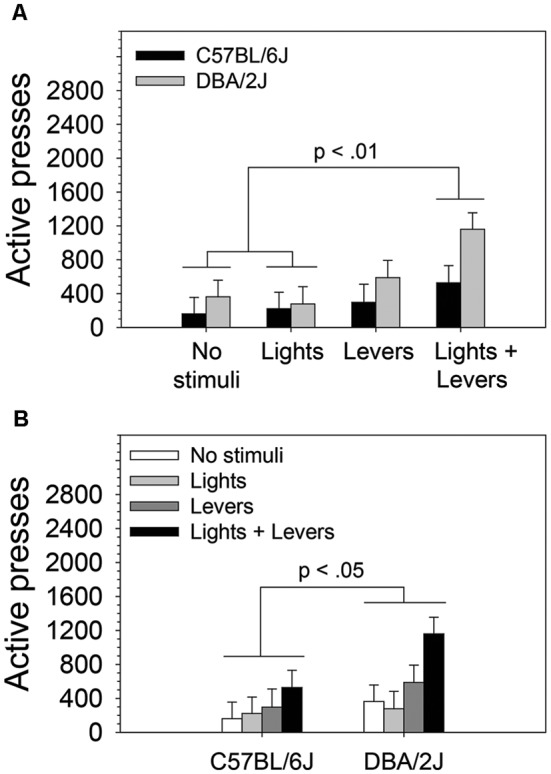
The magnitude of operant sensation seeking varies with mouse strain and is enhanced by stimulus complexity. The effects of stimulus complexity and mouse strain on the magnitude of OSS were assessed in C57BL/6J and DBA/2J mice following 26 days of testing. The dependent measure was the mean of active lever presses on the final four sessions. A two-way ANOVA (stimulus complexity × mouse strain) revealed statistically significant main effects of stimulus complexity (*F*_(3,83)_ = 3.89, *p* = 0.01) and mouse strain (*F*_(1,83)_ = 4.34, *p* = 0.04). The two-way interaction of stimulus complexity and mouse strain was not statistically significant (*F*_(3,83)_ = 0.75, *p* = 0.52). *Post hoc* tests (Fisher’s LSD) for the two statistically significant main effects revealed that **(A)** mice in the *Lights + Levers* condition pressed the active lever significantly more than mice in the *No stimuli* condition (*p* = 0.004) and the *Lights* condition (*p* = 0.004), and **(B)** DBA/2J mice pressed the active lever significantly more (*p* = 0.04) than C57BL/6J mice. Bars represent means. Error bars represent standard errors.

Because we observed statistically significant main effects in the absence of a statistically significant interaction, we performed *post hoc* tests (Fisher’s LSD) to assess the difference between active lever pressing of mice in the *No stimuli* condition*, Lights* condition*, Levers* condition, and *Lights + Levers* condition irrespective of mouse strain (Figure 2A) and (2) the difference between active lever pressing of C57BL/6J and DBA/2J mice irrespective of the level of stimulus complexity (Figure 2B). As illustrated in Figure 2A, mice in the *Lights + Levers* condition pressed the active lever significantly more than mice in the *No stimuli* condition (*p* = 0.004) and the *Lights* condition (*p* = 0.004). As illustrated in Figure 2B, DBA/2J mice pressed the active lever significantly more (*p* = 0.04) than C57BL/6J mice. Collectively, these data indicate that stimulus complexity and mouse strain influence the magnitude of OSS.

### Within-Session Escalation of OSS Is Enhanced by Stimulus Complexity in C57BL/6J and DBA/2J Mice

**Figure 3:**
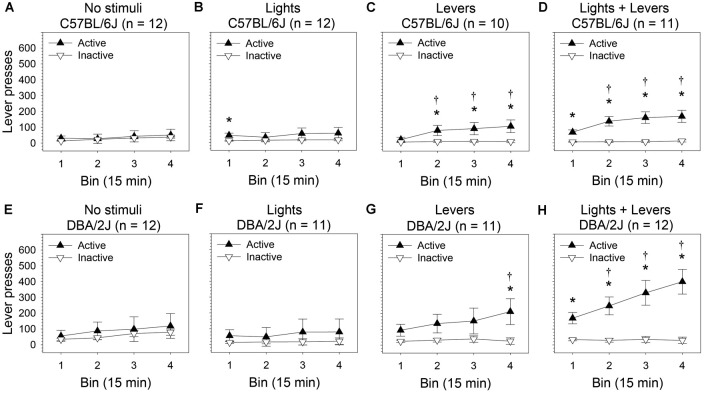
Within-session escalation of OSS is enhanced by stimulus complexity in C57BL/6J and DBA/2J mice. The effect of stimulus complexity on the magnitude of within-session escalation of OSS was assessed in C57BL/6J and DBA/2J mice following 26 days of testing. The number of lever presses (mean of final four sessions) was used as the dependent variable. The relationship between active lever pressing and inactive lever pressing varied significantly across bins as a function of stimulus complexity as indicated by a statistically significant three-way interaction of these factors (three-way ANOVA) in both C57BL/6J mice (*F*_(9,123)_ = 3.23, *p* = 0.01) and DBA/2J mice (*F*_(9,126)_ = 2.92, *p* = 0.009). *Post hoc* tests (Fisher’s LSD) were performed to characterize these relationships:** (A,B,E,F)** Neither C57BL/6J nor DBA/2J mice in the *No stimuli* condition and *Lights* condition escalated active lever pressing across bins. **(C,D,G,H)** In contrast, both C57BL/6J and DBA/2J mice in the *Levers* condition and *Lights + Levers* condition escalated active but not inactive lever pressing across bins. ^†^Bins (2, 3, or 4) on which the number of active lever presses was significantly greater (*p* < 0.05) than the number of active lever presses on Bin 1 within strain and stimulus complexity condition. *Bins on which the number of active lever presses was significantly greater (*p* < 0.05) than the number of inactive lever presses within strain and stimulus complexity condition. Data points represent means. Error bars represent standard errors.

To assess the effects of stimulus complexity on within-session escalation of OSS following 26 days of testing, we used a three-way ANOVA. The number of lever presses was used as the dependent variable (mean of final four sessions). The three independent variables were stimulus complexity (levels: *No stimuli, Lights, Levers, Lights + Levers*), bin (levels: 1–4), and lever (levels: *active, inactive*). Stimulus complexity was a between-subjects variable whereas bin and lever were within-subjects variables. This analysis was performed separately for C57BL/6J mice and DBA/2J mice, and the results for each strain are reported below and illustrated in Figure 3.

#### C57BL/6J Mice

In C57BL/6J mice (Figures 3A–D), we observed statistically significant main effects of bin (*F*_(3,123)_ = 15.75, *p* = 0.00001) and lever (*F*_(1,41)_ = 19.45, *p* = 0.00007). The main effect of stimulus complexity was not significant (*F*_(3,41)_ = 1.34, *p* = 0.27). Most importantly, the three-way interaction of these factors was statistically significant (*F*_(9,123)_ = 3.23, *p* = 0.01) indicating that the relationship between the active lever and the inactive lever changed across bins as a function of stimulus complexity.

Because the three-way interaction for C57BL/6J mice was statistically significant, we performed *post hoc* tests (Fisher’s LSD) for each of the four stimulus conditions. We assessed: (1) the difference between active lever pressing on bin one relative to bins two, three, and four (i.e., escalation of active lever pressing); (2) the difference between inactive lever pressing on bin one relative to bins two, three, and four (i.e., escalation of inactive lever pressing); and (3) the difference between active and inactive lever pressing on each bin. Active but not inactive lever pressing of C57BL/6J mice in the *Levers* condition (Figure 3C) and *Lights + Levers* condition (Figure 3D) escalated significantly across bins (*p* < 0.05 for all tests). Specifically, C57BL/6J mice pressed the active lever significantly more on bins two, three, and four than on the first bin in the *Levers* condition and the *Lights + Levers* condition; active lever pressing was significantly greater than inactive lever pressing on most bins. In contrast, active lever pressing of C57BL/6J mice in the *Lights* condition (Figure 3B) and *No stimuli* condition (Figure 3A) did not escalate significantly across bins. These data reveal a positive causal relationship in C57BL/6J mice between stimulus complexity and within-session escalation of active but not inactive lever pressing in the OSS paradigm.

#### DBA/2J Mice

In DBA/2J mice (Figures 3E–H), we observed statistically significant main effects of bin (*F*_(3,126)_ = 16.04, *p* = 0.000004) and lever (*F*_(1,42)_ = 13.76, *p* = 0.0006). The main effect of stimulus complexity approached significance (*F*_(3,42)_ = 2.26, *p* = 0.09). Most importantly, the three-way interaction of these factors was statistically significant (*F*_(9,126)_ = 2.92, *p* = 0.009) indicating that the relationship between the active lever and the inactive lever changed across bins as a function of stimulus complexity.

Because the three-way interaction for DBA/2J mice was statistically significant, we performed *post hoc* tests (Fisher’s LSD) for each of the four stimulus conditions. We assessed: (1) the difference between active lever pressing on bin one relative to bins two, three, and four (i.e., escalation of active lever pressing); (2) the difference between inactive lever pressing on bin one relative to bins two, three, and four (i.e., escalation of inactive lever pressing); and (3) the difference between active and inactive lever pressing on each bin. Active but not inactive lever pressing of DBA/2J mice in the *Levers* condition (Figure 3G) and *Lights + Levers* condition (Figure 3H) escalated significantly across bins (*p* < 0.05 for all tests). Specifically, DBA/2J mice in the *Lights* + *Levers* condition pressed the active lever significantly more on bins two, three, and four than on the first bin; DBA/2J mice in the *Levers* condition pressed the active lever significantly more on bin four than on the first bin. Active lever pressing of DBA/2J mice was significantly greater than inactive lever pressing on one bin in the *Levers* conditions and on all bins in the *Lights + Levers* condition. In contrast, active lever pressing of DBA/2J mice in the *Lights* condition (Figure 3F) and *No stimuli* condition (Figure 3E) did not escalate significantly across bins and did not differ from inactive lever pressing on any bin. Collectively, these data reveal a positive causal relationship in both C57BL/6J mice and DBA/2J mice between stimulus complexity and within-session escalation of active but not inactive lever pressing in the OSS paradigm.

### The Magnitude of Within-Session Escalation of OSS Is Enhanced by Stimulus Complexity and Mouse Strain

To assess the effects of stimulus complexity and mouse strain on the magnitude of within-session OSS escalation following 26 days of testing, we performed a two-way ANOVA. The difference between the number of active lever presses on the first bin and the final bin (mean of final four sessions) was used as the dependent variable. The two independent variables were stimulus complexity (levels: *No stimuli, Lights, Levers, Lights + Levers*) and mouse strain (levels: C57BL/6J, DBA/2J). Stimulus complexity and strain were between-subjects variables. We observed statistically significant main effects of stimulus complexity (*F*_(3,83)_ = 5.79, *p* = 0.001) and mouse strain (*F*_(1,83)_ = 3.92, *p* = 0.05). The two-way interaction of stimulus complexity and mouse strain was not statistically significant (*F*_(3,83)_ = 0.91, *p* = 0.43).

**Figure 4:**
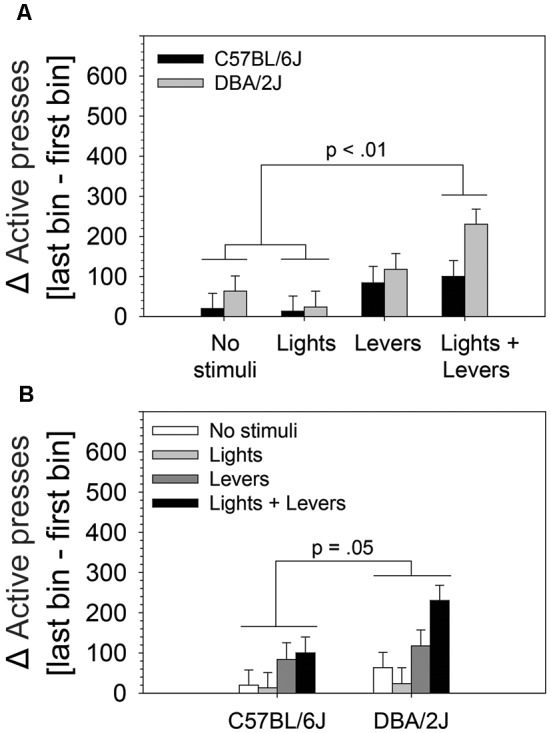
The magnitude of within-session escalation of operant sensation seeking varies with mouse strain and is enhanced by stimulus complexity. The effects of stimulus complexity and mouse strain on the magnitude of within-session escalation of OSS were assessed in C57BL/6J and DBA/2J mice following 26 days of testing. The dependent variable was the mean of active lever presses on the final four sessions. A two-way ANOVA (stimulus complexity × mouse strain) revealed statistically significant main effects of stimulus complexity (*F*_(3,83)_ = 5.79, *p* = 0.001) and mouse strain (*F*_(1,83)_ = 3.92, *p* = 0.05). The two-way interaction of stimulus complexity and mouse strain was not statistically significant (*F*_(3,83)_ = 0.91, *p* = 0.43). *Post hoc* tests (Fisher’s LSD) for the two statistically significant main effects revealed that **(A)** mice in the *Lights + Levers* condition exhibited significantly greater escalation of active lever pressing across bins relative to mice in the *No stimuli* condition (*p* = 0.001) and the *Lights* condition (*p* = 0.0002), and **(B)** DBA/2J mice exhibited significantly greater escalation of active lever pressing across bins relative to C57BL/6J mice (*p* = 0.05). Bars represent means. Error bars represent standard errors.

Because we observed statistically significant main effects in the absence of a statistically significant interaction, we performed *post hoc* tests (Fisher’s LSD) to assess: (1) differences between active lever pressing escalation of mice in the *No stimuli* condition*, Lights* condition*, Levers* condition, and *Lights + Levers* condition irrespective of mouse strain (Figure 4A); and (2) the difference between active lever pressing escalation of C57BL/6J and DBA/2J mice irrespective of the level of stimulus complexity (Figure 4B). As illustrated in Figure 4A, mice in the *Lights + Levers* condition escalated active lever pressing significantly more than mice in the *No stimuli* condition (*p* = 0.001) and the *Lights* condition (*p* = 0.0002). As illustrated in Figure 4B, DBA/2J mice escalated active lever pressing significantly more (*p* = 0.05) than C57BL/6J mice. Collectively, these data indicate that stimulus complexity and mouse strain influence the magnitude of OSS escalation.

## Discussion

In the present study, we used the OSS assay, an operant model of sensation seeking, to test the hypothesis that sensation seeking varies as a function of stimulus complexity and genetic background. We quantified sensation seeking in male mice from the C57BL/6J and DBA/2J inbred strains using three different sensory stimuli that varied in complexity and number of sensory modalities (Table 1). We compared the performance of these mice to control mice that were tested under the same conditions but that did not receive a lever-press contingent sensory stimulus.

This study led to four principal conclusions. First, all sensory stimuli used in the study, regardless of stimulus complexity or number of stimulus modalities, served as reinforcers for C57BL/6J and DBA/2J mice (Figure 1). Second, for both C57BL/6J and DBA/2J mice, sensation seeking for a high complexity multimodal sensory stimulus (*Lights + Levers* condition) was significantly greater than sensation seeking for a low complexity unimodal sensory stimulus (*Lights* condition; Figure 2A). Third, for both C57BL/6J and DBA/2J mice, sensation seeking escalated significantly within-session when a multimodal sensory stimulus of medium or high complexity was used (*Levers* condition and *Lights + Levers* condition, respectively) but not when a unimodal sensory stimulus of low complexity was used (*Lights* condition; Figure 3). Finally, both the magnitude of sensation seeking (Figure 2B) and the magnitude of within-session escalation of sensation seeking (Figure 4B) were significantly greater in mice from the DBA/2J strain relative to mice from the C57BL/6J strain.

### Sensation Seeking Is Positively Related to Stimulus Complexity in C57BL/6J and DBA/2J Mice

In the OSS assay, a significant preference for the active lever was observed on multiple sessions in all stimulus presentation groups but not in the *No stimuli* group (Figure 1). This indicates that sensory stimuli, even those of relatively low complexity and from a single sensory modality, can serve as reinforcers in both C57BL/6J and DBA/2J mice. Moreover, the number of active lever presses increased with stimulus complexity in both strains (Figure 2A), and the number of sessions on which mice discriminated between the active and inactive lever was greatest in the high complexity *Lights + Levers* condition (Figure 1). This indicates that, for both C57BL/6J and DBA/2J mice, the reinforcement magnitude of a sensory stimulus is positively related to the complexity of that stimulus.

The effect of stimulus complexity on OSS in C57BL/6J mice has been studied at least twice before: Barnes and Baron (1961) assessed performance of C57BL/6J mice on an OSS task in which a complex visual pattern (a circle, a square, an x, or a randomly selected pattern from this group) or no pattern was presented on a digital display following a lever press. These authors found that all stimuli were reinforcing relative to no pattern and that lever pressing increased as a function of pattern complexity. In a separate study, Olsen and Winder (2012) assessed performance of C57BL/6J mice on an OSS task in which a purely visual stimulus (a static stimulus light) or a multimodal stimulus (flashing stimulus lights of varying duration and frequency combined with sound from an infusion pump) was presented following a lever press. These authors found that, except for static visual stimuli, all stimuli were reinforcing relative to the absence of lever-press contingent stimulus presentation. They also found that the number of active lever presses increased with the number of stimulus modalities and the degree of stimulus complexity. The C57BL/6J data from the present study are consistent with and lend further support to the findings of Barnes and Baron (1961) and the findings of Olsen and Winder (2012).

### C57BL/6J and DBA/2J Mice Exhibit Within-Session Escalation of Sensation Seeking for High and Medium Complexity But Not Low Complexity Sensory Stimuli

Active but not inactive lever pressing increased significantly across bins during a 60-min OSS testing session when a multimodal sensory stimulus of medium or high complexity was used (Figures 3C,D,G,H). In contrast, active lever pressing was stable across bins for both C57BL/6J and DBA/2J mice when a relatively less complex flashing visual stimulus was used as the reinforcer (Figures 3B,F). To our knowledge, this is the first observation of robust within-session stimulus-complexity dependent escalation of sensation seeking in mice or rats.

Within-session changes in OSS have been extensively described in outbred Sprague–Dawley rats (reviewed in Lloyd et al., 2014). In these studies, well-trained male and female rats exhibit a decrease in FR1 responding over a 30- to 60-min session; this is likely due, at least in part, to the use of a very low complexity sensory stimulus consisting of stimulus light onset. Using C57BL/6J mice as subjects and purely visual stimuli of varying shapes presented on a digital display as a reinforcer, Barnes and Baron (1961) observed a decrease in FR1 responding over an 18-min testing period. Considered together, data from the present study and past OSS studies suggest that within-session escalation of OSS (i.e., sensitization of reinforcer effectiveness), within-session reduction of OSS (i.e., habituation of reinforcer effectiveness), and stable OSS across a session can all be induced through manipulation of the complexity of the sensory stimulus used as the reinforcer.

### Sensation Seeking and Within-Session Escalation of Sensation Seeking Are Significantly Greater in DBA/2J Mice Relative to C57BL/6J Mice

Irrespective of sensory stimulus condition, DBA/2J mice exhibited significantly greater sensation seeking (Figure 2B) and significantly greater within-session escalation of sensation seeking (Figure 4B) when compared to C57BL/6J mice. To our knowledge, the effect of genetic background on sensation seeking and within-session escalation of sensation seeking across multiple levels of stimulus complexity has never been studied in a mouse strain other than C57BL/6J. Comparing the performance of C57BL/6J and DBA/2J strains is useful because they are the founders of the BXD recombinant inbred mouse panel which segregates 6 million C57BL/6J and DBA/2J variants (Peirce et al., 2004; Ashbrook et al., 2019) and can be used to discover genes that drive natural phenotypic variation (Dickson et al., 2016, 2019; Parker et al., 2017). Findings from the current study indicate that the approach used here could be applied to the full BXD recombinant inbred panel to discover genetic mechanisms driving sensation seeking as well as the sensitization and habituation of sensory reinforcer effectiveness.

## Conclusion

In the present study, we have shown that the escalation of OSS, both within and across sessions, is directly influenced by stimulus complexity. Moreover, we have shown that sensation seeking and within-session escalation of sensation seeking are manifested differently in the founder strains of the BXD RI panel. Future studies should incorporate male and female mice to assess the potential influence of sex on these relationships. The ability to easily and rapidly induce escalation or reduction in sensation seeking in genetically distinct mouse strains provides a strong model for discovery of the genetic mechanisms that distinguish controlled from uncontrolled reward-seeking which is a core feature of addiction (American Psychiatric Association, 2013). The study of sensation seeking is directly relevant to addiction because human, mouse and rat studies reveal that sensation seeking and drug and alcohol seeking are driven by shared genetic mechanisms (Zuckerman, 1986; Piazza et al., 1990; Olsen and Winder, 2009; Belin and Deroche-Gamonet, 2012; Flagel et al., 2014; Dickson et al., 2015, 2016, 2019). Therefore, studying the loss of control over sensation seeking can reveal mechanisms driving the loss of control over drug and alcohol seeking. Collectively, findings from the present study and those from previous studies indicate that: (1) a systems genetics approach using the BXD panel will enable discovery of genes underlying sensation seeking and its relationship to stimulus complexity; and (2) the genes discovered in this approach may reveal genetic mechanisms underlying drug and alcohol addiction.

## Data Availability Statement

The datasets generated for this study are available on request to the corresponding author.

## Ethics Statement

The animal study was reviewed and approved by Institutional Animal Care and Use Committee at the University of Memphis.

## Author Contributions

PD designed the experiment which was performed in GM’s lab. PD performed the experiments, analyzed the data, and drafted the manuscript. PD and GM finalized the manuscript for publication.

## Conflict of Interest

The authors declare that the research was conducted in the absence of any commercial or financial relationships that could be construed as a potential conflict of interest.
